# Global Dieting Trends and Seasonality: Social Big-Data Analysis May Be a Useful Tool

**DOI:** 10.3390/nu13041069

**Published:** 2021-03-25

**Authors:** Myung-Bae Park, Ju Mee Wang, Bernard E. Bulwer

**Affiliations:** 1 Department of Gerontal Health and Welfare, Pai Chai University, Daejeon 35345, Korea; 2 The Korean Cardiac Research Foundation, Seoul 04158, Korea; 3 BEB-Noninvasive Cardiovascular Research, Cardiovascular Division, Brigham and Women’s Hospital, Boston, MA 02115, USA

**Keywords:** big-data, diet, weight loss, google, seasonality, cosinor

## Abstract

We explored online search interest in dieting and weight loss using big-data analysis with a view to its potential utility in global obesity prevention efforts. We applied big-data analysis to the global dieting trends collected from Google and Naver search engines from January 2004 to January 2018 using the search term “diet,” in selected six Northern and Southern Hemisphere countries; five Arab and Muslim countries grouped as conservative, semi-conservative, and liberal; and South Korea. Using cosinor analysis to evaluate the periodic flow of time series data, there was seasonality for global search interest in dieting and weight loss (amplitude = 6.94, CI = 5.33~8.56, *p* < 0.000) with highest in January and the lowest in December for both Northern and Southern Hemisphere countries. Seasonal dieting trend in the Arab and Muslim countries was present, but less remarkable (monthly seasonal seasonality, amplitude = 4.07, CI = 2.20~5.95, *p* < 0.000). For South Korea, seasonality was noted on Naver (amplitude = 11.84, CI = 7.62~16.05, *p* < 0.000). Our findings suggest that big-data analysis of social media can be an adjunct in tackling important public health issues like dieting, weight loss, obesity, and food fads, including the optimal timing of interventions.

## 1. Introduction

### 1.1. Big-Data in Public Health

Big-Data is defined as “data sets that are so voluminous and complex” that they overwhelm traditional data analytic methods [1]. The “three Vs”—volume, velocity, and variety–is a popular concept used to describe big-data. This reflects not only just the huge volumes of data but also the speed at which such data is generated and the wide range of data involved. Big-data analytic methods are better suited for analyzing massive datasets in a myriad of rapidly evolving scenarios [2,3].

One of the major advantages of big-data is that it can analyze global data cost-effectively, reliably, and accurately. The Pillbox project of the United States National Library of Medicine (NLM Pillbox) is an often cited example of how healthcare can be improved using big-data [4,5]. Although the NLM Pillbox was shut down in January 2021, similar technologies are being utilized [6], including health projects in other countries [7]. Pillbox served as a large, powerful public service database with information on a wide range of both prescription and over-the-counter (OTC) drugs. It was designed to help users to rapidly identify such drugs based on their ingredients and appearance. It simultaneously delivered and collected information based on user queries, which enhanced convenience, saved costs, and improved consumer safety, among other benefits. Big-data analysis by supercomputers such as IBM Watson that utilize machine learning and artificial intelligence algorithms, can improve diagnosis, minimize errors, and improve care [8].

Another example of Big-data application was Google’s influenza forecast (Google Flu Trends). Massive amounts of data obtained from global online search patterns from dozens of countries generated real-time insights and “nowcasts” about suspected influenza activity worldwide. In 2009, Google predicted the spread of the flu 7–10 days earlier than the U.S. Centers for Disease Control and Prevention based on such online search data about the flu [9]. Several countries, including South Korea, India, and China have compared the predictive value of such data from online searches with actual numbers obtained from traditional public health approaches [10,11,12,13]. Predictive analyses using social big-data can also reliably predict other global seasonal trends, e.g., mental health issues like depression [14,15]. Big-data analyses have limitations, but upgrades and improvements are constantly provided with the aim of improving accuracy and precision [16,17].

### 1.2. Dieting

Dieting and weight loss efforts are global pursuits, considering the known health risks of obesity. Dieting mostly refers to a change in eating habits, but it is often included with increased physical activity or exercise as part of a weight management regimen [18,19]. Obesity, especially central or visceral obesity, is an established risk factor for several diseases, especially cardiovascular disease, type II diabetes, musculoskeletal diseases, and cancer [20,21,22]. Obesity is also a factor in mental health disorders and depression [23,24,25], with potential adverse effects on interpersonal relationships [26].

Global rates of obesity are on the rise, with an obesity epidemic observed in the Arab/Muslim world [27], with seasonal trends observed in dieting and weight loss efforts in Western societies [28,29,30]. Therefore, global health efforts aimed at prevention of obesity are warranted [31,32]. 

With the advent on the Internet and social media, there is a heavy profit-driven fad industry online [33]. Much of this is wholly cosmetic, driven by a heavy emphasis on body image [34,35,36]. Such fads and food trends based on pseudo-science and quackery not only fail to deliver the results promised but also pose a real risk to individual health and well-being [34,37,38]. 

### 1.3. Aims and Goals

We aimed to explore whether the massive amounts of data generated during online search interest in dieting and weight loss could be harnessed, using Big-data analysis, with a view to its potential incorporation in global health obesity prevention efforts. We aimed to explore whether there were seasonal trends, and perhaps an optimal time to potentially target people with online search interests in dieting. In this pursuit, we hypothesized that “*Interest or attempt to explore dieting online would be tend to be seasonal.*” Our study, therefore, (i) examined the time series and seasonality of dieting globally, using social Big-data collected from online portals, and (ii) aimed to suggest timely health intervention strategies based on such findings. 

## 2. Methods

### 2.1. Searching Tools and Keyword

Data was collected through Google web portal (Menlo Park, CA, USA), the most widely used global search engine, with its share of global search averaging 85–90% in most markets, except China [39]. Since January 2004, Google has provided information on time-critical search terms through Google Trends. Such searches can be categorized as searches via the general web, by images, by shopping, or on YouTube, etc. Searches were further categorized and analyzed according to global or individual country statistics. For South Korea, the search engine Naver was added, as its share of the South Korean market was 59% in December 2020, exceeding that of Google [40]. 

This search terms “diet,” “dieting,” and “weight loss” were used, and the monthly Pearson’s correlation coefficient between the three words was 0.946~0.980 (Table 1). Of these, the search volume for “diet” was overwhelmingly high. Since the terms weight loss and weight control could convey other meanings, the search term “diet” was finally selected.

### 2.2. Study Population and Data

Three countries were selected from the Northern Hemisphere (the United States (U.S.), Ireland, and the United Kingdom (UK)) and three from the Southern Hemisphere (South Africa, Australia, and New Zealand), which had the highest search volumes for the search term “diet.” The reference standard was based on the search volumes, where interest in the six selected countries was the highest. As diet and weight loss are also influenced by socioeconomic, cultural, and religious factors [41,42], we also selected five predominantly Arab and Muslim countries, excluding Iraq and Turkey, and categorized them further as (i) conservative, (ii) semi-conservative, and (iii) liberal [43]. South Korea was also studied using the Naver search engine, due to its local dominance as aforementioned. 

### 2.3. Theoretical. Model

Cosinor analysis is a method used to evaluate the periodic flow of time series data as a cosine function. This analysis has been frequently used to analyze body cycles (24 h), such as circadian rhythm [44,45,46]. It is also used to demonstrate the seasonality of differences in blood pressure, stroke incidence, and vitamin D concentration in the 12-month follow-up period [47,48,49]. The model equation is f(t) = M+A cos (ωt + Ø), M is midline estimating statistic of rhythm, A (amplitude) is the distance from the highest point, ω is the number of vibrations, Ø (acrophase) is the time from the reference time to the first matching A, t is the time unit [50].

Therefore, when the A is large, the change of the value is large, i.e., the search volume fluctuates more depending on the season. In addition, when acrophore and time are short, it means the period (wave) is lacking in our study. 

### 2.4. Statistical Analyses

The data was analyzed by standardizing the number of periods with the longest search period as 100 and the lowest as 0. In this study, we used web-search data from January 2004 to February 2021, and the time unit being 1 month. On the other hand, South Korea used data starting from February 2016 to February 2021, since no data from Naver was available prior to February 2016. The alpha level was set at t *p* > 0.05. Data was reported as mean ± SD (standard deviation). Additionally, we reported amplitude and 95% confidence intervals (95% CI) for within-group comparisons. The correlations between search terms were analyzed using Pearson’s coefficients. We used statistical software R version 3.6.2, and the package “cosinor” in R for data analysis.

## 3. Results

### 3.1. Descriptive Statistics 

The combined results from the two Hemispheres were the highest in January (86.0 ± 16.6) and the lowest in December (41.5 ± 10.6) for on-line diet searches. Most diet searches were performed in January for both the Northern and the Southern Hemispheres. In contrast, December was the month with the lowest on-line searches for diet. For the Arab/Muslim countries, April was the leading month for on-line diet searches (32.3 ± 20.9), with October (25.3 ± 15.8) being the lowest for on-line diet searches. Based on socio-religious categories, for conservative Arab/Muslim countries, their highest online search month was April (40.5 ± 13.9), for semi-conservative countries was June (22.8 ± 24.6), and for liberal countries was March (43.1 ± 18.3). The month with the lowest search interest in diet for conservative countries was March (27.8 ± 13.1), for semi-conservative countries was January (17.0 ± 16.5) and for liberal Arab/Muslim countries was December (30.8 ± 11.4). For South Korea, when searching on Korean internet portals in Korean language only, the results were much higher than those obtained when using English. Therefore, we included both Google and Naver searches for South Korea, where the results based on Google searches were highest in January (23.2 ± 22.4) and lowest in December (16.3 ± 13.5). The results for Naver searches were highest in March (62.2 ± 20.2) and lowest in December (36.2 ± 5.6) (Table 2).

### 3.2. Cosinor Analysis 

**Figure 1:**
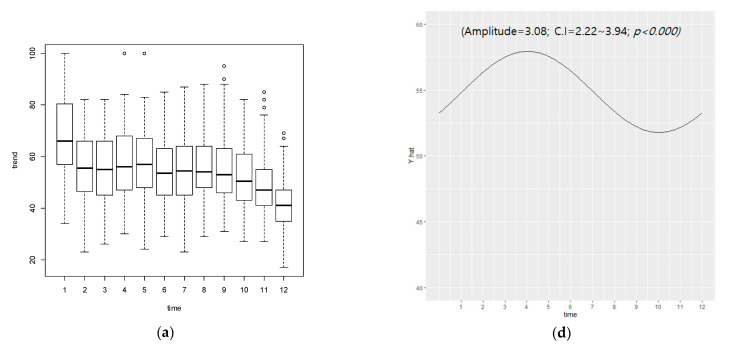

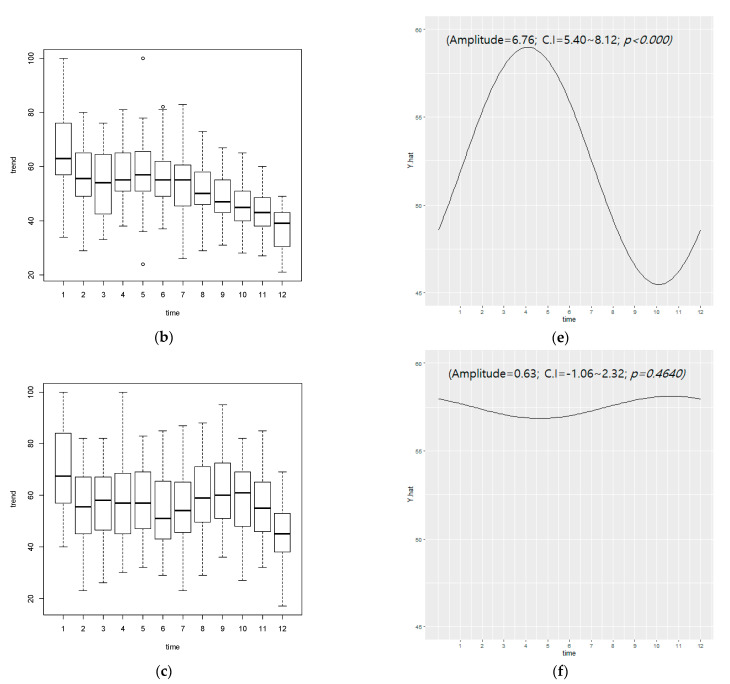
Monthly search volume and cycle for diet in Northern-Southern Hampshire. Search volume of (**a**) six countries with highest search volume, (**b**) three Northern, and (**c**) three Southern countries. Cycle by cosinor analysis of (**d**) six countries with highest search volume, (**e**) three Northern, and (**f**) three Southern countries. CI: 95% confidence interval; Unit of time is 1 month.; *p*-value < 0.05 corresponds to the statistical significance of seasonal periodicity of diet interest in each group.

As a result of cosinor analysis of data from the Northern and Southern Hemispheres, there was seasonality for on-line diet searches (amplitude = 3.08, confidence interval; CI = 2.22~3.94, *p* < 0.000), with diet searches being the highest in January and the lowest in December. As a result of analyzing only the Northern Hemisphere, seasonality for on-line diet searches (amplitude = 6.76, CI = 5.40~8.12, *p* < 0.000) was highest in January and lowest was in December. In the Southern Hemisphere, seasonal variation of the curve was not statistically significant (amplitude = 0.63, CI= −1.06~2.32, *p* = 0.464) as shown in Figure 1, i.e., no seasonality for on-line diet searches was observed in the Southern Hemispheres, Australia, and New Zealand. 

**Figure 2:**
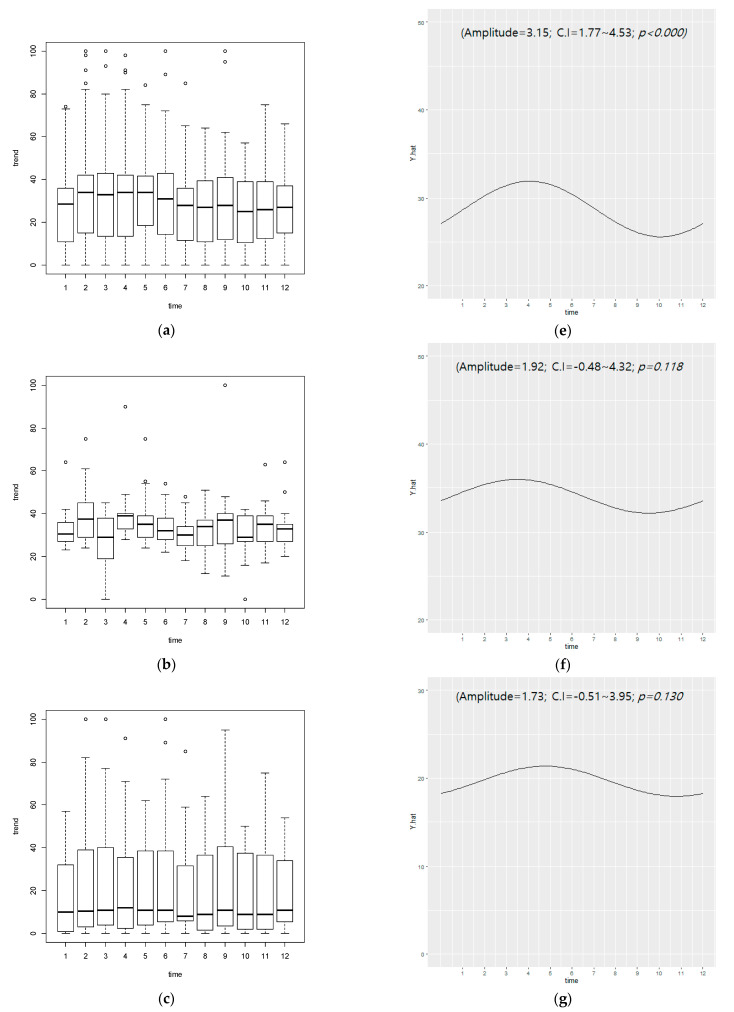

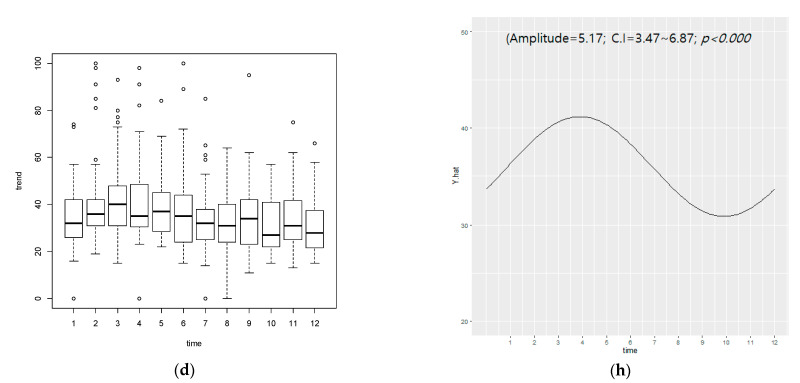
Monthly search volume and cycle for diet in five Arab and Muslim countries. Search volumes of (**a**) the five primarily Arab and Muslim countries in our study and the subcategories (**b**) conservative (**c**) semi-conservative, and (**d**) liberal. Cycle by cosinor analysis of (**e**) five Arab and Muslim countries search volume, (**f**) conservative countries, (**g**) semi-conservative countries, (**h**) liberal countries. C.I: 95% confidence interval; Unit of time is 1 month.; *p*-value < 0.05 corresponds to the statistical significance of seasonal periodicity of diet interest in each group.

When data from the five Arab/Muslim countries were integrated, monthly seasonal periodicity for on-line diet searches was observed (amplitude = 3.15, CI = 1.77~4.53, *p* < 0.000). For seasonal periodicity on a monthly basis in conservative countries like Saudi Arabia, amplitude = 1.92, CI = −0.48~4.32, *p* = 0.118, for semi-conservative countries, amplitude = 1.73, CI = −0.51~3.95, *p* = 0.13), and for liberal countries, amplitude = 5.17, CI = 3.47~6.87, *p* < 0.000. The trends for the monthly curve of these three groups were similar (Figure 2). There appears to be a degree of seasonality of diet interests. However, the amplitude was smaller than the Northern Hemisphere average. 

**Figure 3:**
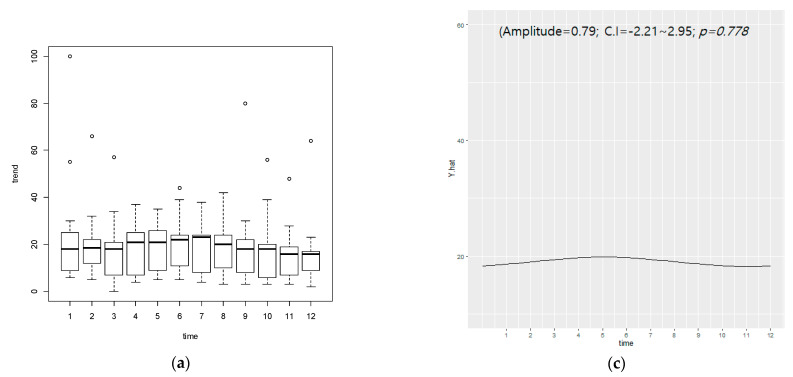

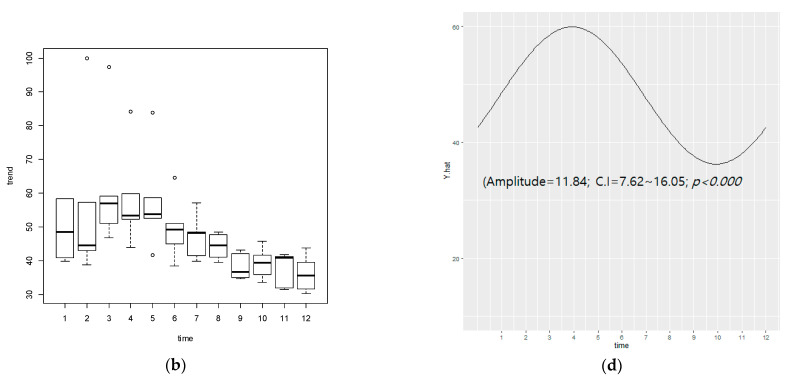
Monthly search volume and cycle for diet in South Korea. Search volume of (**a**) Google Trends and (**b**) Naver data. Cycle by cosinor analysis of (**c**) Google Trends and (**d**) Naver data. CI: 95% confidence interval; unit of time is 1 month.; *p*-value < 0.05 corresponds to the statistical significance of seasonal periodicity of diet interest in each group.

For South Korea, the seasonal periodicity of monthly data was not seen (amplitude = 0.79, CI = −2.21~2.95, *p* = 0.778) in the Google data, but seasonal periodicity was statistically significant in data using Naver (amplitude = 11.84, CI = 7.62~16.05, *p* < *0*.000) (Figure 3). 

## 4. Discussion

Public interest in dieting for the purposes of weight management is near universal, but there is little published evidence to support this. Our study aimed to explore and compare global seasonality in diet and dieting trends using social Big-data analysis. 

The search term “diet” used in this context is synonymous with dieting for weight loss. Our search terms included “diet,” “dieting,” and “weight loss”; the monthly correlation coefficient between the three words was 0.946~0.980. Of these, the search volume for “diet” was overwhelmingly high. In addition, “diet” was highly correlated with weight loss (r = 0.975, *p* < 0.000). Therefore, although the word “diet” is a broad term when used alone, in the context of our search, phrases like “going on a diet” are now synonymous with dieting, especially in reference to global obesity and weight loss efforts. Therefore, we considered that the search term “diet” by the selected countries in our study to be representative of global interest in dieting for weight loss.

Global interests in this subject based on big-data from online searches could become a useful addition to public health intervention strategies for obesity management, weight loss, and associated diseases. Obesity is an important global public health challenge, as it is a major risk factor for cardiovascular and chronic diseases, with major impact on morbidity, mortality, and health care costs. Effective management of obesity includes prevention of premature death and disability, reducing the economic burden of disease, and the promotion of healthy diets and lifestyles [31,51,52]. 

With respect to the promotion of healthy diet and lifestyle, our aim was to analyze interest in global dieting and weight loss trends, being cognizant that sociocultural, societal, and traditional practices could potentially play a role. Since dieting and weight loss pursuits are of global interest, with the Internet as a major portal for disseminating information and advertisements, we considered Big-data analysis ideal for studying such large amounts of data on global dieting practices and trends [53,54,55]. No attempt was made to exclude fad diets and weight loss programs, even though these also have potential health risks, including increased risks of eating disorders and mental health problems, including stress, anxiety, and depression [34,56,57,58,59,60,61,62,63,64]. 

In this study, we found that the search volume for dieting in the Northern and Southern Hemispheres was the highest in January, which coincides with the New Year, where people traditionally make New Year’s resolutions following the Christmas holidays. On the other hand, for the predominantly Arab and Muslim countries, the highest search volumes were in April. For South Korea, the highest search volume was observed in February.

On cosinor analysis, which analyzes periodic trends, online search interest in dieting in the Northern Hemisphere was statistically significantly seasonal, but for the Southern Hemisphere, it was not.

Studies using cosinor analysis tend to show opposite dieting trends of Southern compared to Northern Hemisphere countries [49,65], probably reflecting the divergent seasons. The data on global seasonal trends in dieting is apparently limited, but studies on weight changes in three major countries, including Japan, the United States, and Germany showed a sharp increase from December, with the greatest increase in weight in early January, just after the Christmas holiday [66,67]. 

In this study, the search volumes of both Northern and Southern Hemispheres were the highest in January. Overall, search interest reached its peak before summer (April) in the Northern Hemisphere and November in the Southern Hemisphere [68,69,70]. For predominantly Arab and Muslim countries, seasonality was not striking, and the magnitude was smaller than that observed for the Northern Hemisphere, with April being the highest point in the periodic rate. Seasonality tended to be a bit more pronounced in the liberal Arab and Muslim countries, compared to their more conservative counterparts. 

Finally, in South Korea, data from Naver showed seasonality, with April being the peak month for online diet searches, with the trend of rhythm being similar to that of the Northern Hemisphere. However, there was no statistically significant seasonality in the data from Google, which may be a reflection of the lower percentage of Google searches on this topic in South Korea. Considering this skew, Google searchers in South Korea on the subject of dieting was most likely unrepresentative. 

Although our study is exploratory, our Big-data analyses suggest the potential for role for seasonal emphasis on weight control programs. More cost-effective health awareness and prevention weight loss strategies could harness the power of online Big-data analyses and real-time “nowcasting,” for optimal timing of public health interventions for obesity. In the same way, that marketing strategists use such Big-data to target consumers, so, public health authorities too could utilize Big-data for optimal timing of public education and intervention programs.

### Strengths and Limitations

The authors are unaware of any previous studies to analyze the global seasonality of diets using social big-data. Big-data analysis of seasonal dieting trends is rather easy to access and analyze and, therefore, potentially more cost effective. This approach can also hold relevance to other areas of public health. 

Our study has some limitations. First, we conducted the keywords search terms in English only, which is less representative for countries that do not use English as their primary search language, such as in South Korea and in the Arab and Muslim majority of countries we studied. It may therefore be more accurate to include searches using the preferred language of such countries. Second, it is difficult or near impossible to examine the individual characteristics of each person who performed each search, without breaching social media confidentiality or other agreements. Third, we could not accurately predict the actual figures by analyzing the search volume using web-based methods only.

## 5. Conclusions

Big-data analysis can reliably analyze the metadata trends such as global seasonal patterns such as search interests in dieting and weight loss. In our study, some degree of seasonal patterns emerged. Weight management and weight loss strategies could consider such trends for optimal timing of their health promotion and intervention strategies. Big-data analytics, including artificial intelligence algorithms, can be harnessed to provide cost-effective insights, as well as optimal approaches and timing of global health promotion and intervention programs.

## Figures and Tables

**Table 1:** Associations between search terms diet, dieting, and weight loss.

	Diet	Dieting	Weight Loss
**Diet**	1.000	0.980 <0.000	0.975 <0.000
**Dieting**	0.980 <0.000	1.000	0.946 <0.000
**Weight loss**	0.975 <0.000	0.946 <0.000	1.000

Correlations were presented using Pearson’s coefficients with *p*-values; correlations are at statistically significant level at *p*-value < 0.05 and indicates having the associations between search terms diet, dieting, and weight loss.

**Table 2:** Comparison of the search volume of diet by month among study countries.

Month	Six Countries with Highest Search Volume Mean (SD)			Arab and Muslim Countries Mean (SD)				South Korea Mean (SD)	
	Overall	Northern Hemisphere	Southern Hemisphere	Overall	Conservative	Semi-Conservative	Liberal	Google	Naver
January	68.0 (16.6)↑	66.3 (15.8)↑	69.8 (16.8)↑	26.6 (16.5)	33.1 (9.4)	17.0 (16.5)↓	34.1 (13.3)	23.2 (22.4)↑	49.2 (9.0)
February	56.4 (13.0)	56.4 (11.7)	56.4 (14.3)	32.2 (21.6)	39.3 (13.3)	21.0 (21.9)	40.9 (18.6)	20.3 (13.1)	56.7 (25.2)
March	54.9 (12.9)	53.8 (11.9)	56.1 (13.9)	31.9 (22.3)	27.8 (13.1)↓	22.0 (23.5)	43.1 (18.3)↑	17.7 (13.5)	62.2 (20.2)↑
April	57.1 (13.0)	56.8 (10.5)	57.3 (15.2)	32.3 (20.9)↑	40.5 (13.9)↑	21.0 (21.1)	40.9 (17.3)	17.8 (10.1)	58.7 (15.3)
May	57.6 (13.1)	57.9 (12.4)	57.3 (13.9)	30.8 (18.2)	37.9 (12.9)	20.2 (18.6)	39.1 (13.3)	18.8 (9.5)	58.1 (15.7)
June	54.8 (12.4)	55.6 (10.7)	54.0 (13.9)	31.5 (22.0)	34.5 (9.1)	22.8 (24.6)↑	39.1 (19.3)	21.1 (10.8)	49.7 (9.6)
July	54.8 (12.8)	53.7 (11.6)	55.9 (14.0)	26.7 (17.7)	30.4 (8.8)	18.6 (19.5)	33.6 (14.7)	20.1 (11.1)	47.1 (6.8)
August	54.9 (13.5)	50.7 (11.1)	59.1 (14.4)	26.1 (16.7)	31.4 (10.4)	18.4 (18.5)	32.1 (13.3)	19.2 (10.7)	44.3 (4.0)
September	55.4 (13.8)	48.3 (7.9)	62.5 (14.9)	29.2 (20.0)	36.3 (19.3)	21.1 (21.4)	34.9 (15.8)	19.9 (17.3)	38.3 (4.0)
October	52.3 (13.0)	45.4 (7.3)	59.3 (13.9)	25.3 (15.8)↓	29.6 (11.1)	17.8 (18.0)	31.3 (11.1)	17.5 (13.6)	39.3 (4.7)
November	49.8 (12.3)	43.7 (7.5)	55.9 (13.2)	26.6 (16.8)	34.3 (107)	18.1 (18.6)	32.6 (12.5)	15.8 (10.8)	37.5 (5.3)
December	41.5 (10.6) ↑	37.2 (7.7)↓	45.8 (11.5)↓	25.7 (15.2)	33.6 (10.6)	18.0 (16.4)	30.8 (11.4)↓	16.3 (13.5)↓	36.2 (5.6)↓

SD standard deviation; higher the search volume indicates the closer to 100 and the lower the search volume indicates the closer to 0; ↑ indicates highest month for on-line diet searches, ↓ indicates lowest month for on-line diet searches.

## Data Availability

All data can be used and analyzed through ‘https://trends.google.com/’ accessed on 14 March 2021 and ‘https://datalab.naver.com/’ accessed on 14 March 2021.
